# Faecal inoculations alter the gastrointestinal microbiome and allow dietary expansion in a wild specialist herbivore, the koala

**DOI:** 10.1186/s42523-019-0008-0

**Published:** 2019-08-21

**Authors:** Michaela D. J. Blyton, Rochelle M. Soo, Desley Whisson, Karen J. Marsh, Jack Pascoe, Mark Le Pla, William Foley, Philip Hugenholtz, Ben D. Moore

**Affiliations:** 1 Hawkesbury Institute for the Environment, Western Sydney University, Sydney, New South Wales 2753 Australia; 2 Australian Centre for Ecogenomics, University of Queensland, Brisbane, Queensland 4067 Australia; 3 School of Life & Environmental Sciences, Deakin University, Melbourne, Victoria 2134 Australia; 4 Ecology and Evolution, The Australian National University, Canberra, ACT 2601 Australia; 5 Conservation Ecology Centre, Cape Otway, Victoria 3233 Australia

**Keywords:** Koala, Gastrointestinal, Microbiome, Diet, *Eucalyptus*, Faecal transplant, *E. obliqua*, *E. viminalis*

## Abstract

**Background:**

Differences between individuals in their gastrointestinal microbiomes can lead to variation in their ability to persist on particular diets. Koalas are dietary specialists, feeding almost exclusively on *Eucalyptus* foliage but many individuals will not feed on particular *Eucalyptus* species that are adequate food for other individuals, even when facing starvation. We undertook a faecal inoculation experiment to test whether a koala’s gastrointestinal (GI) microbiome influences their diet. Wild-caught koalas that initially fed on the preferred manna gum (*Eucalyptus viminalis)* were brought into captivity and orally inoculated with encapsulated material derived from faeces from koalas feeding on either the less preferred messmate (*E. obliqua*; treatment) or manna gum (control).

**Results:**

The gastrointestinal microbiomes of wild koalas feeding primarily on manna gum were distinct from those feeding primarily on messmate. We found that the gastrointestinal microbiomes of koalas were unresponsive to dietary changes because the control koalas’ GI microbiomes did not change even when the nocturnal koalas were fed exclusively on messmate overnight. We showed that faecal inoculations can assist the GI microbiomes of koalas to change as the treatment koalas’ GI microbiomes became more similar to those of wild koalas feeding on messmate. There was no overall difference between the control and treatment koalas in the quantity of messmate they consumed. However, the greater the change in the koalas’ GI microbiomes, the more messmate they consumed after the inoculations had established.

**Conclusions:**

The results suggest that dietary changes can only lead to changes in the GI microbiomes of koalas if the appropriate microbial species are present, and/or that the koala gastrointestinal microbiome influences diet selection.

**Electronic supplementary material:**

The online version of this article (10.1186/s42523-019-0008-0) contains supplementary material, which is available to authorized users.

## Background

Dietary niche has long been recognised as a driving factor in determining species’ distributions. Yet the role that a species’ gastrointestinal microbiome plays in shaping that niche has only recently been considered, with little empirical evidence available from wild study systems [1]. The gastrointestinal (GI) microbiome is intimately involved in mammalian digestion and nutrition [2–5]. Different animal species have distinct microbial assemblies that are thought to be specially adapted to the digestion of the host’s diet [6]. Indeed, even within a species, differences in the GI microbiome may assist the host to persist on particular diets, for example, by detoxifying plant secondary metabolites (PSMs) found at high abundances in those diets [7–9]. Therefore, in addition to constraints imposed by factors such as food availability, social structure and life history, an animal’s diet may be restricted if they do not possess an appropriate microbial assemblage. Such dietary niche restriction could have important ramifications for a species’ persistence in different habitats. Additionally, the capacity for an animal’s GI microbiome to adapt to changes in the host’s diet may affect conservation initiatives including translocations and captive breeding, where animals may be faced with a sudden change in diet. Thus there can be wide benefits from gaining an understanding of the conditions under which the GI microbiome can or cannot adapt to host diet and how such adaptation impacts the host.

One species for which these questions are of immediate relevance is the koala (*Phascolarctos cinereus*). The koala is a specialised folivore of the tree genus *Eucalyptus*, with individual koalas feeding on between one and ten species, making it one of the most specialised mammalian herbivores [10]. Koala diet choice is strongly influenced by nutritional content (particularly protein) and by plant secondary chemistry, which influence the digestibility and toxicity of the diet [11, 12]. On Cape Otway, Australia, in 2013, the population density of koalas was such that they over-browsed their preferred food tree, manna gum (*E. viminalis*), causing widespread defoliation and tree mortality [13]. This, in turn, led to more than 70% of the koalas dying due to starvation and compassionate euthanasia [13], and prompted ongoing intervention by wildlife authorities that involved translocation of over 400 healthy koalas in 2015 [14]. Critically, even though koalas were starving, most did not feed on adjacent stands of another abundant *Eucalyptus* species, messmate (*E. obliqua*). However, there were a small number of koalas that were resident in the messmate forest and for which messmate was the preferred diet. One hypothesis for why the majority of koalas failed to shift diets is that they were unable to subsist on or were dissuaded from feeding on the messmate foliage because their GI microbiomes were not appropriate and were unable to adapt.

The GI microbiome of the koala is thought to primarily play a role in fermenting dietary fibre and other refractory materials [15–18]. Microbial fermentation occurs in the enormously expanded hindgut (caecum and proximal colon) of koalas after nutrients such as simple sugars and amino acids (protein) have been absorbed in the small intestine [19]. In contrast, in herbivorous foregut-fermenting animals such as cattle (*Bos* spp.) the GI microbiome breaks down highly refractory material in expanded areas of the gut prior to the site of true acid digestion [20]. These animals then acquire nutrients from the digestion of the microbes and their metabolic products. Thus, foregut-fermenting animals are likely to be affected by the abilities of their GI microbiome’s to digest and detoxify all dietary components. In contrast, the GI microbiome may interact with the dietary niche of hindgut-fermenting animals primarily through particular dietary fractions such as fibre.

The GI microbiomes of koalas that feed on messmate have been found to differ from those that feed on manna gum [21]. Nutritionally, manna gum foliage has more available protein and although relatively highly digestible for a *Eucalyptus*, is defended by a specific group of secondary metabolites [21, 22]. Messmate foliage contains less available protein and higher concentrations of fibre [21]. Thus, koalas feeding on messmate may rely on the short chain fatty acids produced by microbial fermentation of fibre to a greater extent than do koalas feeding on manna gum. Reflecting this, the GI microbiomes of koalas feeding on messmate have greater relative abundances of fibrolytic bacteria, have a greater diversity of gene functions and are enriched for genes involved the degradation of more resistant structural components such as xylan [21, 23]. The GI microbiomes of koalas eating messmate also appear to be optimised to utilise different complex carbohydrate (fibre) sources to those utilised by koalas feeding on manna gum [23]. This suggests that the gut microbiomes of koalas can be finely tuned to optimally digest the particular species of *Eucalyptus* and that adaptation of the GI microbiome may be required in the event of dietary change.

The capacity of an animal’s GI microbiome to adjust when required, to a particular diet in part depends on whether the required change can be achieved by a shift in the relative abundances of existing taxa or requires additional microbes not present in the starting community. Certainly, changes in relative abundance can often be achieved rapidly with a change in diet and can optimise the function of the GI microbiome for the digestive challenge faced [24]. However, in some cases the introduction of new, particular microbes is required [8, 25] and adaption to a new diet will depend on whether the required microbes can be readily acquired from conspecifics, the environment or the diet itself. Such horizontal transmission is known to occur in many species [24, 26], yet it may be restricted to particular types of bacteria, and vertical transmission (from one host generation to the next) may predominate [27], limiting the ability of the GI microbiome to adapt [28]. Koalas acquire their GI microbiomes as joeys by ingestion of maternal caecal contents (“pap”) around the time of pouch emergence [29]. Therefore, there is likely to be strong vertical transmission of the GI microbiome in this species and a koala’s GI microbiome is likely to be similar to that of their mother. The extent of horizontal transmission is unknown, however, given that koalas are solitary and arboreal, conspecific transmission and thus the GI microbiomes’ adaptive potential may be limited.

To test the hypothesis that koalas’ GI microbiomes limit their diet, we undertook an experiment in which koalas brought into captivity from manna gum forest at Cape Otway were inoculated with faecally-derived microbes either from wild koalas feeding predominately on messmate (treatment) or manna gum (control). During the experiment koalas were encouraged to feed on messmate by restricting their access to other food sources. In doing so we aimed to gain insights into whether the koala’s GI microbiome is capable of responding to a diet change; to ascertain if faecal inoculations assist the GI microbiome in responding to a change in diet; and to test whether a koala’s GI microbiome influences diet selection. Oral faecal inoculation have previously been used in humans to successfully treat *Clostridium difficile* infection [30]. They have also been used with or without antibiotics to alter the GI microbiomes of several rodent species for research purposes [8, 31]. Our experiment provides the first assessment, to our knowledge, of the effectiveness of oral faecal inoculations in introducing and establishing enteric microbes into the GI microbiome of a wild solely hindgut-fermenting mammal that is an extreme dietary specialist. We demonstrate that our method of encasing the inoculum in acid-resistant capsules can successfully introduce certain microbes providing avenues for future probiotic development and allowing us to successfully test our ecological questions.

## Results

### The GI microbiomes of koalas feeding on messmate or manna gum

**Fig. 1:**
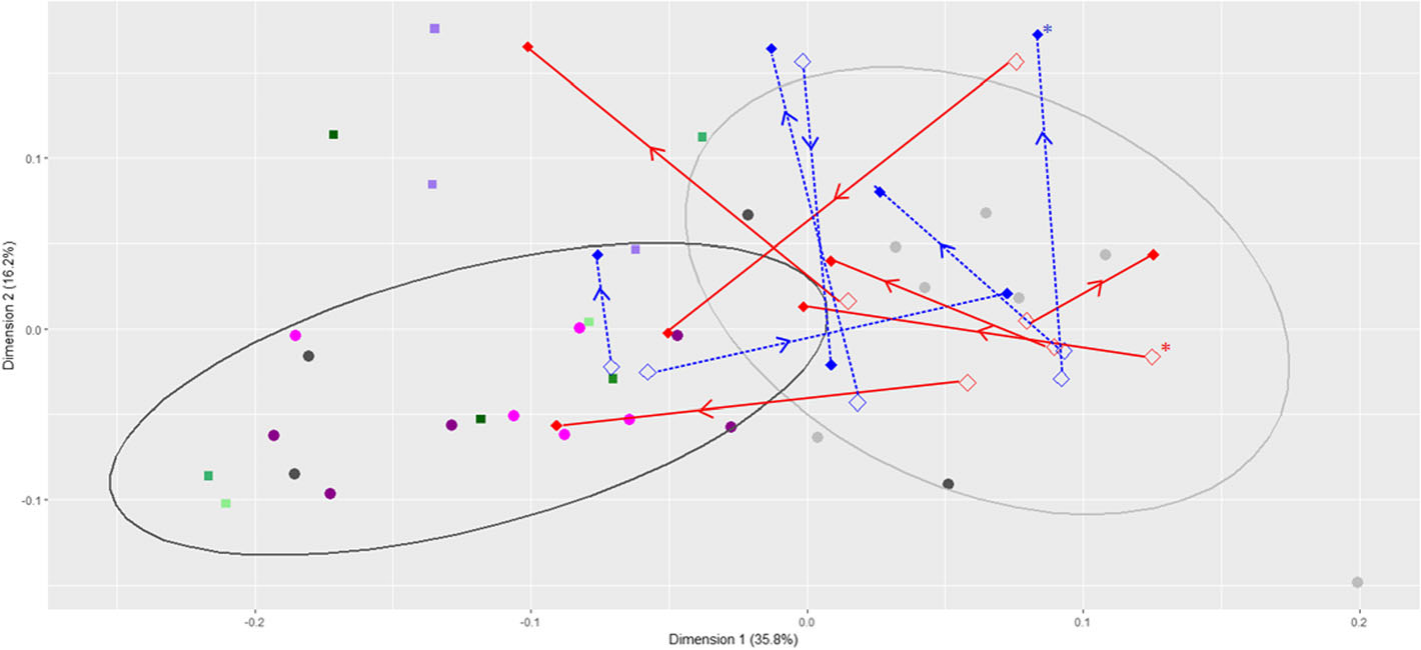
First two dimensions of the principal components analysis of the weighted Unifrac distances. Light grey and dark grey circles represent koalas located in manna gum and messmate forest respectively. With the corresponding 90% ellipses shown (ellipses were calculated assuming a multivariate t-distribution from 7 samples from 5 koalas captured in manna gum forest and 12 samples from 8 koalas, including donor koalas, located in messmate forest). Treatment koalas are shown in red, while, control koalas are shown in blue. Open diamonds represent the koalas’ GI microbiomes at capture (with the exception of one treatment koala where the capture sample was not available and instead the sample prior to messmate introduction was used, indicated by the *), while closed diamonds show the koalas’ GI microbiomes at the conclusion of the experiment (except for the control koala released early for which their final sample, 9 days after inoculation is shown; indicated with the *). Lines join points from the same captive koala with the direction their GI microbiomes shifted over the experiment indicated by arrows. Dark purple circles indicate the GI microbiomes of the treatment donor koalas for cohort 1, while, pink circles indicate those for cohort 2. Very light green, light green, medium green and dark green squares indicate the bacterial, coarse, solute and fine layers respectively. Light purple squares indicate the inoculum

Our results confirm the previous findings of Brice and colleagues [21] that showed that the GI microbiomes of koalas feeding on messmate differ from those of koalas feeding on manna gum based on 16 s rRNA profiles (PERMANOVA: F = 15.378, *p* < 0.001, R^2^ = 0.49). The GI microbiomes of koalas found in manna gum forest in this study were found to separate from those of koalas found in messmate forest on dimension 1 (PC1) of the principal components analysis (t = − 5.288, *p* < 0.001; Fig. 1). However, some overlap between the groups was seen, suggesting that the diets and GI microbiomes of koalas at Cape Otway form a continuum and do not represent two populations feeding exclusively on messmate or manna gum. This conclusion is supported by the observation that the two treatment donor koalas (ID: L and R) that were found in messmate trees on only 50 and 61% of occasions had GI microbiomes that were intermediate between those of koalas feeding on manna gum and other koalas feeding on messmate.

**Fig. 2:**
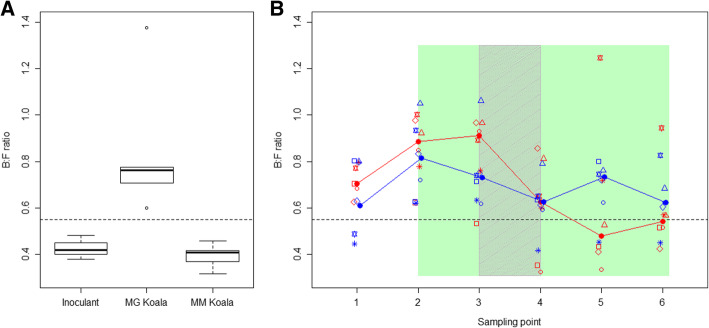
Bacteroidetes to Firmicutes (B:F) ratios of koalas. **a** boxplot of the Bacteroidetes to Firmicutes (B:F) ratio of koalas found in manna gum (*n* = 5; MG Koala) and messmate forest (i.e. donor koalas from MG2, n = 5; MM Koala) and that of the inoculant given to the treatment koalas. **b** scatter plot of the Bacteroidetes to Firmicutes (B:F) ratio of the treatment (red) and control (blue) captive koalas over time. Closed circles joined by lines indicate the median values for each treatment. Open symbols indicate B:F ratio for each individual, with different symbols indicating different individuals. The koalas received messmate over the period indicated in green with the inoculation period indicated by the grey shading. Sampling point 1 = Capture; 2 = immediately prior to the introduction of messmate; 3 = three days after the introduction of messmate; 4 = immediately after the completion of the inoculations; 5 = nine days after the completion of the inoculations; and 6 = 18 d after the completion of the inoculations

There was a significant difference between the GI microbiomes of koalas from manna gum forest and the GI microbiomes of koalas from messmate forest in the ratio of the relative abundances of the Bacteroidetes and Firmicutes phyla (B:F mean manna gum koala = 0.77, mean messmate koala = 0.39, Fig. 2, *p* < 0.001). Additionally, all manna gum koalas had a B:F ratio greater than 0.55, while, all messmate koalas had a ratio lower than this. The two donor koalas that were found in messmate on between 50 and 61% of occasions (IDs: L and R) had B:F ratios closer to those of koalas feeding on manna gum (L = 0.53, R = 0.58). There was a very strong correlation between PC1 score and the B:F ratio (Spearman rank correlation = 0.97; *p* < 0.001).

There were no significant differences in GI microbiome alpha diversity between koalas found in manna gum and messmate forest (Chao index, Shannon diversity and feature count: *p* > 0.7). However, there was a significant negative correlation between PC1 score and Shannon diversity (R = -0.37, *p* < 0.001), although richness (Chao index and feature count) was not correlated with PC1 score. The PC2 score was significantly positively correlated with all measures of alpha diversity (R: Shannon = 0.4, Chao = 0.25, and feature count = 0.3; *p* < 0.01).

Indicator species analysis identified 26 microbial features that were significantly associated with a messmate diet (Table 1). This included 16 features that were assigned to the phylum Firmicutes, of which all but two were assigned to the order *Clostridiales*. Within the order *Clostridiales* ten of the features belonged to the family *Lachnospiraceae*, while the remaining four belonged to the family *Ruminoccacease*. Twelve features were only detected in messmate koalas, although each was only detected in 37.5 to 50% of messmate donor koalas. The combined relative abundance of these microbial features was on average 10.5% in the messmate koalas, while it was significantly lower at 1% in the manna gum koalas (z = − 3.329, *p* = 0.001). Seven features were found at greater than 1% average relative abundance in the messmate koalas (Table 1).

**Table 1:** List of features that were significantly associated with a messmate diet as revealed by indicator species analysis

Taxonomic designation of feature	%^1^	Donors^2^	A^3^	B^4^	Stat^5^	P^6^	Established In^7^
D0_Bacteria; D1_Bacteroidetes; D2_Bacteroidia; D3_Bacteroidales;							
D4_Bacteroidaceae; D5_Bacteroides	2.90	5	0.786	0.875	0.829	0.021	0
D4_Prevotellaceae	0.45	2	1.000	0.375	0.612	0.039	0
D0_Bacteria; D1_Cyanobacteria; D2_Melainabacteria;							
D3_Gastranaerophilales;							
D4_uncultured bacterium	0.51	6	0.826	0.750	0.787	0.028	3
D4_uncultured rumen bacterium	0.14	2	1.000	0.375	0.612	0.035	0
D0_Bacteria; D1_Firmicutes	0.13	2	0.984	0.375	0.607	0.036	0
D0_Bacteria; D1_Firmicutes							
D2_Bacilli; D3_Lactobacillales; D4_Streptococcaceae;							
D5_Streptococcus	0.02	4	0.803	0.625	0.709	0.041	0
D2_Clostridia; D3_Clostridiales;							
D4_Lachnospiraceae	0.40	4	0.984	0.500	0.702	0.018	1
D4_Lachnospiraceae	0.04	2	1.000	0.375	0.612	0.038	0
D4_Lachnospiraceae	0.06	2	1.000	0.375	0.612	0.037	1
D4_Lachnospiraceae	0.20	3	1.000	0.375	0.612	0.038	0
D4_Lachnospiraceae	0.04	3	1.000	0.375	0.612	0.033	0
D4_Lachnospiraceae	0.05	4	1.000	0.500	0.707	0.010	0
D4_Lachnospiraceae;							
D5_Hungatella	0.06	3	0.969	0.375	0.603	0.038	0
D5_Lachnospiraceae NK4A136 group	2.85	4	0.957	0.625	0.773	0.008	2
D5_Lachnospiraceae NK4A136 group	2.43	6	0.993	0.750	0.863	0.001	2
D5_Lachnospiraceae UCG-001	4.15	3	0.981	0.500	0.700	0.044	6
D4_Ruminococcaceae	0.05	3	0.921	0.500	0.679	0.035	0
D4_Ruminococcaceae	1.83	4	0.973	0.500	0.697	0.049	0
D4_Ruminococcaceae							
D5_Ruminococcaceae UCG-010	1.66	4	0.984	0.750	0.859	0.001	0
D5_Ruminococcaceae UCG-014; D6_uncultured bacterium	0.02	2	1.000	0.375	0.612	0.033	0
D0_Bacteria; D1_Proteobacteria; D2_Gammaproteobacteria;							
D3_Pasteurellales; D4_Pasteurellaceae	0.02	2	1.000	0.375	0.612	0.037	0
D0_Bacteria; D1_Verrucomicrobia; D2_Verrucomicrobiae;							
D3_Verrucomicrobiales; D4_Verrucomicrobiaceae;							
D5_Akkermansia; D6_uncultured bacterium	1.74	3	1.000	0.375	0.612	0.038	6
Unassigned^8^	0.02	7	0.933	0.750	0.837	0.001	2
Unassigned^8^	0.23	3	0.970	0.375	0.603	0.039	1
Unassigned^8^	0.03	4	1.000	0.500	0.707	0.011	0
Unassigned^8^	0.01	4	1.000	0.500	0.707	0.011	0

1. Mean relative abundance of feature when it was detected in the GI microbiomes of koalas feeding on messmate

2. Donors = the number of donor koalas the feature was found in

3. A = the probability that a sample came from a messmate koala given the feature was found. Only taxa with A > 80% were considered as indicator taxa

4. B = the probability of finding that feature in a koala feeding on messmate. Only taxa with B > 35% were considered as indicator taxa

5. stat = √ (A x B)

6. *p* value of ‘stat’ was determined by 1000 random permutations of the data

7. Established In = the number of treatment koalas the feature was found to establish in

8. Unassigned = features that did not fall within a recognised taxonomic classification

### Treatment inoculant

We assessed the microbial composition of the treatment inoculum that was made from the bacterial and fine particle layers formed during the centrifugation of the donor faecal material (Additional file 2: Figure S1) and delivered to the koalas’ orally via acid resistant capsules. Both the treatment inoculum and the layers clustered with the treatment donor faecal samples on PC1 and had B:F ratios of less than 0.55 (Figs. 1 and 2). However, the inoculum samples and two of the layer samples (the fine particle layer from the single koala and the coarse particle layer from a mixed koala faecal sample) had higher values on PC2 than the treatment donor faecal samples (Fig. 1). This may be attributed to these samples having higher alpha diversity because they were produced from the pooled faecal samples of five different koalas. Shannon diversity, Chao1 alpha diversity and the number of detected features were all significantly higher in the inoculum samples than in the faecal samples from koalas found in messmate forest (Wilcoxon rank sum test: *p* = 0.009, 0.036 and 0.009 respectively).

### Success of the faecal inoculations

Ten of the twelve koalas captured for the faecal inoculation experiment had GI microbiomes that, at the time of capture, resembled those of manna gum koalas based on 16 s rRNA profiles. The GI microbiomes of these koalas had high scores on PC1 (Fig. 1) and a B:F ratio greater than 0.55 (Fig. 2). The remaining two koalas (IDs: V and W) had GI microbiomes at capture that clustered with the treatment (messmate) donor koalas on PC1 (Fig. 1) and had B:F ratios of less than 0.55. However, both these koalas had low relative abundances of the indicator features at the beginning of the experiment (2 and 0.1%) compared to the messmate koalas. While these koalas were captured in manna gum forest, it is possible that they also fed on some messmate. Both these koalas were randomly assigned to the control group prior to GI microbiome characterisation.

**Fig. 3:**
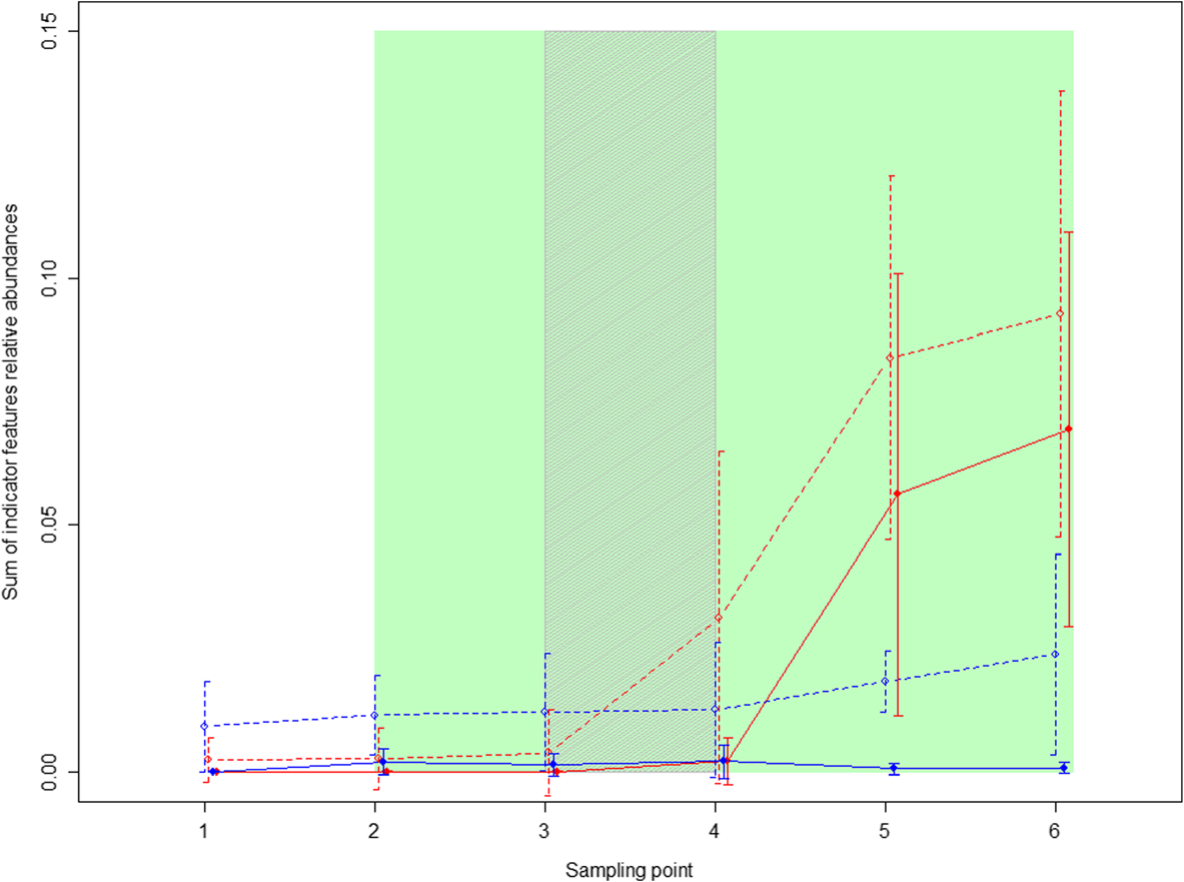
Combined relative abundance of indicator species. Open circles and dotted lines represent all indicator features, while, closed circles and solid lines represent those features for which A = 1. Treatment koalas are shown in red, while, control koalas are in blue. Points joined by lines indicate the mean values for each treatment, while, the error bars indicate the standard deviation. The koalas received messmate over the period indicated in green with the inoculation period indicated by the grey shading. Sampling point 1 = Capture; 2 = immediately prior to the introduction of messmate; 3 = three days after the introduction of messmate; 4 = immediately after the completion of the inoculations; 5 = nine days after the completion of the inoculations; and 6 = 18 d after the completion of the inoculations

The indicator features that were only found in messmate koalas (i.e. A = 1) were not detected in the treatment koalas at capture but were detected and increased in relative abundance after the inoculations (Fig. 3). The pattern was similar for the remaining indicator features, except that they were detected at low frequency in two koalas at capture. The indicator features for which A = 1 were detected at low frequency in the control koalas prior to the inoculations, however, neither these features nor the other indicator features increased in relative abundance over the experiment (Fig. 3). The indicator features were detected at combined abundances between 7.8 and 14% post-inoculation in the treatment koalas. By contrast, the maximum relative abundance of indicator features expected to be detected in the faeces due to washout in the absence of proliferation would be 0.09% (based on the following information: i. The indicator features represented on average 6.7% of the microbial community in the treatment inoculum; ii. Each koala received two capsules or a maximum of 1470 mg of inoculum per day for nine days; and iii. The average digesta pool size for a 6 kg koala is approximately 1 kg [32]). This suggests that at least some microbial species were successfully introduced and at least temporarily established in the treatment koalas’ GI microbiomes. There were no significant difference between the cohorts in the abundance of the indicator species after the inoculations (time points 5 and 6) in either the treatment or control groups (t-test, *p* > 0.8).

The increased combined relative abundance of the indictor features in the treatment koalas can primarily be attributed to two features. The first of these belonged to the family *Akkermansia*, which was only detected in 37.5% of the donors but established and increased in abundance in all treatment koalas. The second was a member of the genus *Lachnospiraceae* UCG-001. This feature was found in 50% of donors, was the most abundant indicator feature (at 4.5% in messmate donors) and also established in all treatment koalas. There were also two additional features belonging to the genus *Lachnospiraceae* NK4A136 that had average relative abundances of greater than 2.5% in the donors and were each transferred to two treatment koalas (ID: E,D and G). A microbial feature belonging to the order *Gastranaerophilales* (phylum: Cyanobacteria, class: *Melainabacteria*) was transferred to three treatment koalas (ID: D, E and G). Two of the remaining three features that were found at greater than 1% relative abundance in the donor koalas both belonged to the family *Ruminococcaceae* and were not transferred to any treatment koalas.

There was no significant change in the alpha diversity of the koalas’ GI microbiomes between the koalas’ capture and their initial presentation with messmate (Chao1: t = 1.272, *p* = 0.209; Number of microbial groups: t = 1.4, *p* = 0.168, Shannon diversity: t = 1.20, *p* = 0.237), demonstrating that captivity did not influence the diversity of the koalas’ GI microbiomes. In fact, there was no significant change in GI microbiome richness in either the treatment or control koalas over the course of the experiment (Chao1: *F* = 0.04, *p* = 0.947; Number of microbial groups: *F* = 0.304, *p* = 0.583) and this did not differ between the cohorts (Chao1: *F* = 1.05, *p* = 0.309; Number of microbial groups: *F* = 0.86, *p* = 0.358). However, Shannon diversity values increased in both the treatment and control koalas after the inoculations (i.e. timepoints 4,5 & 6 had higher Shannon Diversity than time points 1,2 &3), although this increase was only significant in cohort MG1 (MG1; *t* = − 4.92, *p* < 0.001; MG2; *t* = − 0.67, *p* < 0.504). This was likely due to the introduction of additional microbes to the GI microbiomes of these koalas via the inoculations.

**Fig. 4:**
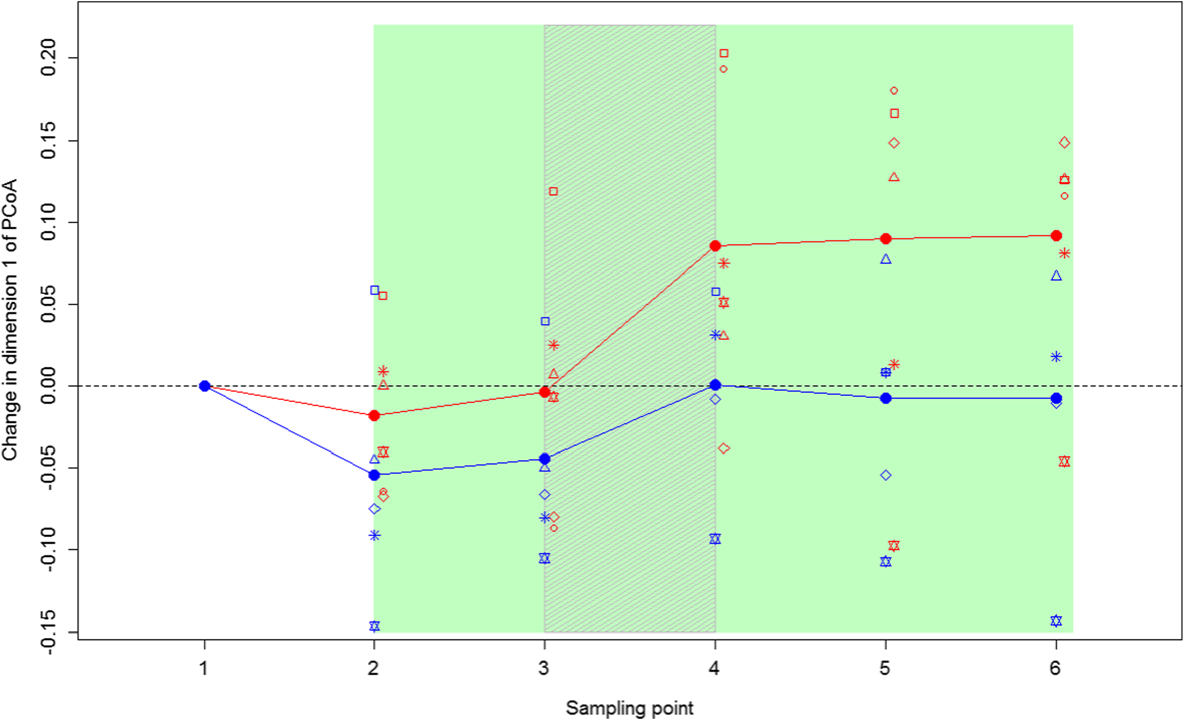
Change in PC1 score of the treatment and control koalas’ GI microbiomes over the study. Treatment koalas are shown in red, while, control koalas are in blue. Closed circles joined by lines indicate the mean values for each treatment. Open symbols indicate the GI microbiome change for each individual, with different symbols indicating different individuals. The koalas received messmate over the period indicated in green with the inoculation period indicated by the grey shading. Sampling point 1 = Capture; 2 = immediately prior to the introduction of messmate; 3 = three days after the introduction of messmate; 4 = immediately after the completion of the inoculations; 5 = nine days after the completion of the inoculations; and 6 = 18 d after the completion of the inoculations

The position of the treatment koalas’ GI microbiomes on PC1 shifted towards the location of the messmate koalas (donor koalas) over the course of the experiment (Figs. 1 and 4). This change was significantly greater than that seen in the control koalas (*t* = − 2.55, *p* = 0.029), with the control koalas on average maintaining similar scores on PC1 after the faecal inoculations (Fig. 4). The magnitude of this change did not differ between the cohorts (*F* = 0.063, *p* = 0.81), however, the magnitude of change did vary between individual treatment koalas (Figs. 1 and 4). This variation in response combined with variation between koalas in their capture GI microbiomes meant that there was no significant difference between the treatment and control groups in the location of their GI microbiomes on PC1 at the conclusion of the experiment (*t* = − 0.49, *p* = 0.417). These results were confirmed by the B:F ratio findings. The B:F ratio of the treatment koalas decreased after the inoculations in both cohorts (comparison of timepoints 1–3 to 4–6; Fig. 2). This decrease was significantly different to the control group (change measured as difference between timepoints 1 and 6 in B:F ratio; *t* = 2.55, *p* = 0.029).

### Total dry matter intake

Total daily dry matter intake (TDMI) ranged from 151 g to 349 g between koalas, with manna gum average daily intake (ADI) increasing with capture body weight (*t* = 3.973, *p* = 0.002; adjusted R^2^ = 0.573). TDMI was found to vary significantly between the different phases of the experiment (*F* = 5.25, *p* < 0.001)*.* TDMI prior to the introduction of messmate (average = 249 g) was significantly higher than during the inoculations (average = 207 g; t = 4.01, p < 0.001) or during the establishment phase (10–18 after the inoculations; average = 237 g; t = 2.10, *p* = 0.037). TDMI was also significantly higher during the washout phase (1–9 days after the inoculations; average = 251 g) than during the inoculations (t = − 3.80, p < 0.001). The more the GI microbiomes of koalas changed over the study to resemble those of messmate koalas, the lower their TDMI was during the inoculations (change in the Bacteroidetes to Firmicutes (B:F) ratio: *F* = 18.06, *p* = 0.013; the change in PC1 scores: *F* = 32.27, *p* = 0.004). There was also a trend for treatment koalas to have lower TDMIs during the inoculations compared to control koalas (*p* = 0.07). After the faecal inoculations (washout and establishment phases), TDMI decreased in those koalas that had microbiomes that did not change to resemble those of messmate koalas, while TDMI remained steady for those koalas that had GI microbiomes that changed (interaction term between the change in B:F ratio and Day: *F* = 8.87, *p* = 0.003; interaction term between PC1 scores and Day: *F* = 7.01, *p* = 0.009).

### Intake of messmate prior to the inoculations

**Fig. 5:**
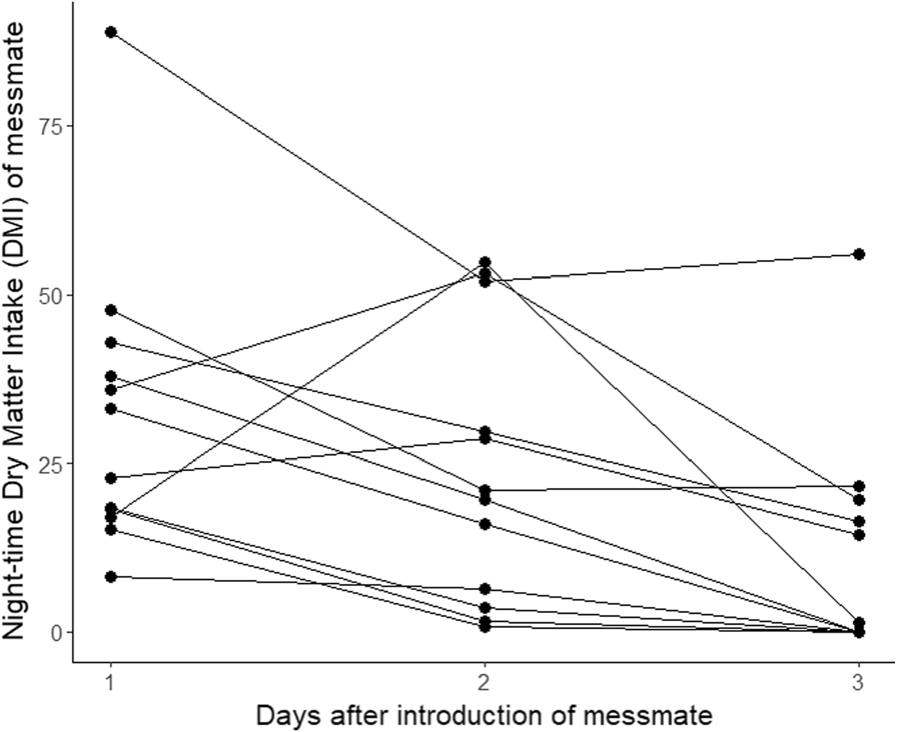
Night-time dry matter intake (grams) of messmate by koalas immediately after introduction of messmate. Points indicate individual koala intakes; lines connect intakes by the same koala

Messmate was introduced to the captive koalas after a period of habituation to captivity, during which time they were fed exclusively on manna gum. We found that the quantity of messmate eaten by the koalas prior to the faecal inoculations decreased over the first three days after it was first introduced (*F* = − 15.411, *p* = 0.029; Fig. 5). There were also significant differences between individual koalas in the amount of messmate that they ate (*F* = 7.415, *p* < 0.001). Neither the koalas’ GI microbiomes at capture nor immediately prior to the introduction of messmate significantly explained messmate intake prior to the inoculations (t = 0.044 and 0.110 respectively, *p* > 0.9). Manna gum average daily intake (ADI) and cohort were also not significant explanatory variables (*p* = 0.752 and 0.378 respectively).

### Intake of messmate during and after the inoculations

After the commencement of the 9 days of faecal inoculations, messmate comprised on average 28.4% of the koalas’ daily dry matter intakes (range = 13.5 to 47.7%). Most of the koalas’ messmate consumption occurred at night when they did not have access to manna gum. We found no overall difference between the treatment and control groups in their night-time intake of messmate (NMI) or in the proportion of their TDMIs that were messmate (PM) over the entire period (27 d) after the first faecal inoculation (NMI: *t* = 0.119, *p* = 0.909; PM: *F =* 0.05, *p* = 0.836). Nor did we find any interaction effect between the experimental group and the number of days after the first faecal inoculation (NMI: *t* = 0.557, *p* = 0.578; PM: *F =* 1.67, *p* = 0.199), indicating that messmate intake did not change over time in the treatment group in a way that differed from the control group. These findings were confirmed by our analysis of each phase of the experiment (i.e. during the faecal inoculations; washout: 1–9 d after the faecal inoculations; and post-establishment: 10–18 d after the inoculations).

**Fig. 6:**
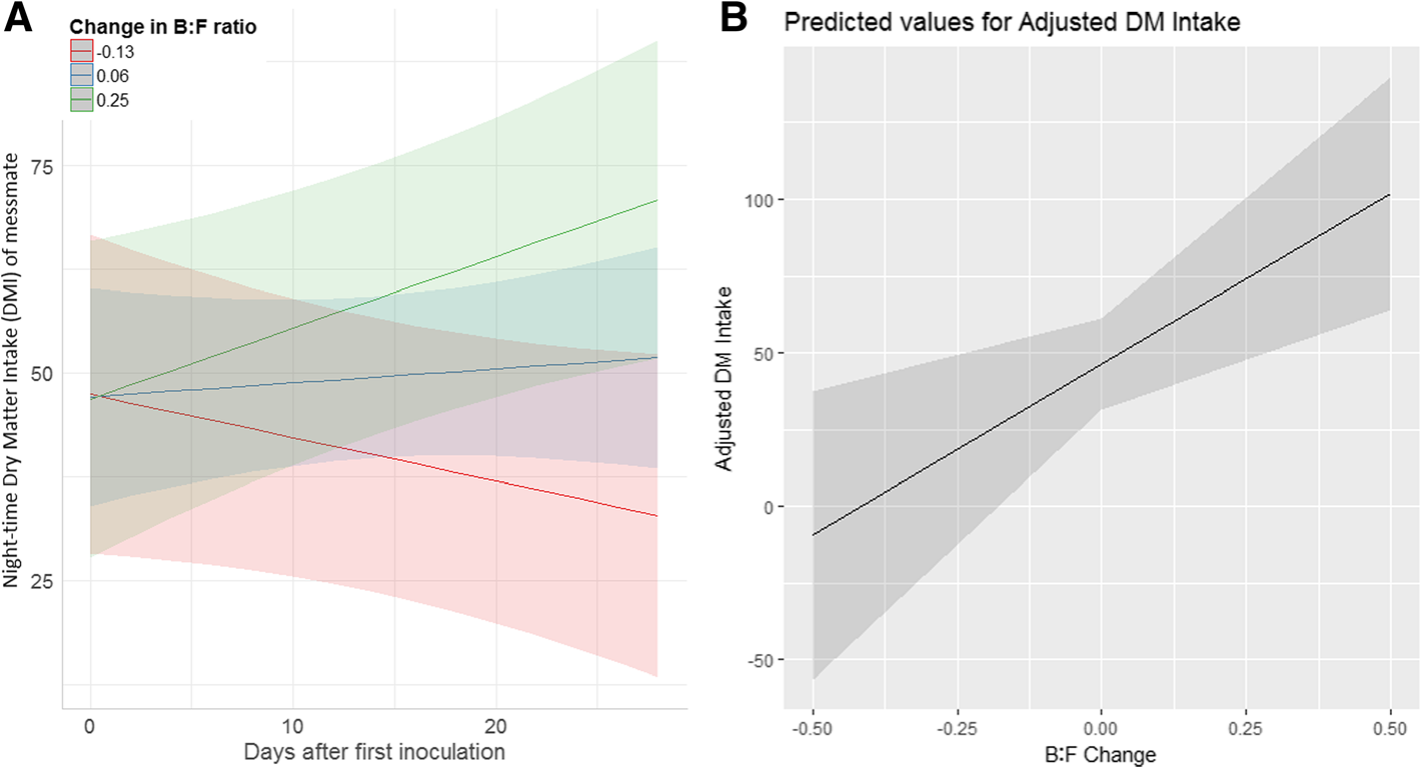
Predicted night-time messmate DMI (g) change in response to the extent of koala GI microbiome change. **a**) shows change over the entire study and **b**) shows the change after the completion of the inoculations. GI microbiome change represented as the change in the Bacteroidetes to Firmicutes ratio, with negative values indicating a shift towards Bacteroidetes and positive values indicating a shift towards Firmicutes. Shading represents 90% confidence intervals. Predictions taken from the final linear mixed effects models

However, night-time messmate intake and messmate proportion were found to significantly increase over time in koalas when their GI microbiomes changed over the study to more closely resemble those of messmate koalas, while messmate intake remained stable or decreased over time in koalas when the measured aspects of their GI microbiomes did not change to more closely resemble those of messmate koalas (interaction term between the change in B:F ratio and Day: NMI: t = 3.702, *p* < 0.001, PM: *F* = 5.01, *p* = 0.027; interaction term between the change in PC1 scores and Day: NMI: *t* = − 2.101, *p* = 0.037, PM: *F* = 4.533, *p* = 0.035; Fig. 6a). This meant that when assessing the different phases of the experiment separately, we found that messmate intake during the last 9 d of the experiment (10–18 d after the inoculations) was significantly higher in koalas that had a greater shift in their GI microbiomes towards an assemblage resembling that of messmate koalas (the change in the B:F ratio: *t* = − 3.30, *p* = 0.010; the change in PC1 scores: *t* = − 2.32, *p* = 0.044; Fig. 6b). However, the extent of GI microbiome change did not explain messmate intake during the inoculations or during the washout phase. The proportion of TDMI that was messmate was also significantly higher after the inoculations (1–18 days) in koalas that had a greater extent of change in their B:F ratios (*F* = 5.647, *p* = 0.040), though the koalas’ GI microbiome positions on the first axis of the PCoA were not significant (*F* = 2.18, *p* = 0.1719).

The koalas’ GI microbiomes both immediately prior to a phase and after a phase were equally highly significant predictors of messmate intake (B:F ratio, before: *t* = − 4.055, *p* < 0.001, after: *t* = − 4.090, *p* < 0.001; PC1 scores, before: *t* = − 3.890, *p* < 0.001, after: *t* = − 3.884, *p* < 0.001; delta AIC: B:F ratio = 0.201,  PC1 scores= 0.068), while the koala’s GI microbiomes at capture were not (PC1 scores: *t* = − 0.522, *p* = 0.618).

When assessing the different phases of the experiment separately, we also found that messmate intake decreased during the period that the faecal inoculations were administered and that this decrease was greater in the treatment koalas than in the control koalas (interaction term between Day and Treatment: *t* = − 2.558, *p* = 0.013). However, messmate intake was seen to increase immediately after the inoculations were concluded (mean adjusted dry matter messmate intake during inoculations = 42.3 g; after inoculations = 57.2; *t =* 2.569; *p* = 0.026).

The koalas’ night-time messmate intake during and after the inoculations was also higher in koalas that had higher pre-inoculation messmate intake and/or higher manna gum ADI (manna gum ADI: *t* = 2.449, *p* = 0.038; pre-inoculation messmate intake: *t* = 2.5, *p* = 0.033). There was no difference between cohorts in messmate intake (*t* = 0.951, *p* = 0.362).

## Discussion

Numerous studies have shown that the GI microbiome can be shaped by host diet [3, 33] and that in some instances the GI microbiome can rapidly adapt to short-term diet changes [24]. However, in hindgut fermenting herbivores where the material entering the caecum is likely to be primarily lignified fibre and other refractory materials [5], the effect of diet on GI microbiome composition may be more restricted. Further, there is also evidence that some aspects of the GI microbiome, including the enterotype of humans, can be resilient to short term diet changes [34]. We have shown that the koala GI microbiome appears to be relatively unresponsive to dietary changes over a period of a month. This is likely to exceed the period of time that an animal would form a behavioural aversion to an inappropriate food source in the wild ([29, 50]). Our study also demonstrates that faecal inoculations can assist the GI microbiomes of koalas to change with a shift in diet. The GI microbiomes of the treatment koalas in our experiment became more similar to those of messmate koalas over the course of the study and appeared to stabilise between nine and eighteen days after the completion of the inoculations. By contrast, the control koalas’ GI microbiomes did not change in the same way. This was despite both treatment and control koalas shifting from a diet solely composed of manna gum to one containing greater than 25% messmate. In further support of this conclusion, we observed that one of the control koalas (ID = U) consumed a comparatively high proportion of messmate (36.5% of TDMI), yet his GI microbiome resembled that of a manna gum koala on capture and did nt become more similar to that of the messmate koalas over the study.

There are two non-mutually exclusive explanations as to why the amount of messmate the koalas consumed was associated with the extent of change in the koalas’ GI microbiomes and why this shift was mainly seen in the treatment koalas. The first is that the introduction of microbes associated with a messmate diet may have enabled the koalas’ GI microbiomes to change (as discussed above), however, a change in the overall GI microbiome composition only occurred in those koalas that fed on messmate (as this selected for microbes that were well adapted for a messmate diet). In support of this explanation, the GI microbiome of one of the treatment koalas (ID = X) did not shift to resemble that of a messmate koala, despite the successful introduction of the indicator species. This koala consumed comparatively small amounts of messmate prior to and after the inoculations (13.5% of TDMI). This suggests that both a change in diet and the presence of the appropriate microbes is required for the GI microbiome to change in this system. Interestingly, this does not appear to be the case in all systems. For instance, the use of probiotics in mice can alter the GI microbiome in the absence of dietary change [35]. Experiments in which koalas are inoculated with faecal material from messmate koalas but not fed messmate could be carried out to test this explanation.

The second explanation we propose to explain our findings is that the inoculations allowed the GI microbiomes to change and that this change allowed the koala to increase its intake of messmate. The finding that the intake of messmate increased over time in the koalas with greater GI microbiome change suggests that this explanation is plausible. It also suggests that there may be a lag in the koalas’ diet preferences after a change in their GI microbiome composition. However, given the naturally circular relationship between the GI microbiome and diet, it is difficult to separate the two suggested explanations.

In the koala, elucidation of the functions and identity of particular microbes that facilitate digestion of messmate is difficult as the proportion of messmate consumed varies between individuals and the GI microbiomes of these koalas form a continuum. Koalas in the wild that feed on both messmate and manna gum were found to exhibit intermediate GI microbiomes and in one instance did not possess the indicator features (koala ID = R). This suggests that changes in the relative abundance of ubiquitous taxa may provide some adaptation to messmate in the diet. Whether these koalas could persist on messmate alone over an extended period of time is unknown.

By focussing on individuals with near-pure diets we were able to identify the presence of indicator features that were associated with a diet of messmate. The fact that a change in the GI microbiome was only observed in the treatment koalas that were administered these indicator features suggests that particular microbial taxa may be crucial for koalas to subsist solely or predominately on messmate. In particular, members of the family *Lachnospiraceae* and *Ruminococcaceae* were associated with a messmate diet. Brice and colleagues [21] found that koalas feeding on messmate possessed higher abundances of these families and that *Ruminococcaceae* genomes possessed more enzymatic genes targeting the degradation of recalcitrant cellulose than members of the genera *Parabacteroides* (found at high abundance in manna gum GI microbiomes). *Lachnospiraceae* are primarily associated with the GI tracts of mammals [36] and both families have previously been identified as fibrolytic [37]. This suggests that these taxa may assist koalas to acquire sufficient energy from the messmate foliage through improved fibre fermentation. Yet, it should be recognised that both koalas feeding on messmate and manna gum possess members of these families. Therefore, further work is needed to identify the indicator microbes to a finer taxonomic resolution and characterise their unique functional characteristics. Particular attention should be given to the genera *Lachnospiraceae* NK4A136 and *Lachnospiraceae* UCG-001 as these were found to transfer to the treatment koalas and were very rare or absent from koalas eating manna gum.

It appears that the fibre content and composition of messmate and manna gum leaves may be critically related to the koalas’ GI microbiomes and their ability or willingness to feed on messmate. There may also be other factors, independent of the microbiome, that influence koalas’ dietary preferences, such as koala age and tooth wear, or differing endogenous capacities for digestion or absorption, detoxification, metabolism and excretion of plant secondary metabolites. One factor that could conceivably limit messmate feeding is the differing secondary metabolite compositions of the two *Eucalyptus* species. Manna gum, contains formylated phloroglucinol compounds (FPCs) in its leaves [22], while messmate contains unsubstituted B-ring flavanones (UBFs; [38]). Both of these groups of compounds have been found to deter feeding by koalas (Marsh et al. unpublished; [39]). Yet the extent to which they interact with the koala GI microbiome is unknown as most terpenes and phenolic compounds are absorbed by the koala prior to the hindgut [40]. In contrast, foregut fermenters like sheep, cattle and (to a lesser extent) woodrats (*Neomtoma* spp.) may benefit more from microbial metabolism of ingested toxins (Foley et al. 1999, Dearing et al. 2005). Tannins are another group of compounds that are at higher abundances in messmate and some bind to dietary protein, preventing its digestion in the small intestine [41]. Tannins also bind to endogenous proteins including mucin, likely increasing protein loss [42], and to microbial enzyme complexes, reducing function [43]. At least some of these tannin protein complexes reach the hindgut of other failovers fed *Eucalyptus* leaf [44]. The koala GI microbiome sometimes contains bacteria that can degrade these tannin-protein complexes [45–47]. However, these bacteria are found at low relative abundances [17, 18] and were not detected in this study. Additionally, as amino acids are only absorbed in the small intestine the koala cannot utilise the amino acids liberated by bacteria in the hindgut [5]. Therefore, it is unclear whether or how any interactions between the secondary metabolites of *Eucalyptus* species and the koala hindgut microbiome impact koala nutrition or diet selection.

In other study systems, the GI microbiome has been directly shown to influence diet selection through a single identifiable secondary metabolite or toxin that can be broken down by known microbial species that inhabits an enlarged foregut. For instance, Australian cattle eat *Leucaena leucocephala* only when bacteria that degrade mimosine are introduced to the rumen [7, 48]. Furthermore, when the foregut pouches of woodrats (*Neotoma* spp.) are inoculated with microbes from animals eating juniper or oxalate-rich cactus species they are able to maintain themselves on the corresponding diet whereas before the inoculation, they eat little and lose weight [8, 9].

Our findings provide some support for the hypothesis that an inappropriate GI microbiome may in part explain why koalas that were starving after defoliating manna gum in 2013 did not switch their diet to messmate despite its availability to a large proportion of the population. Further, our observation that the koalas ate more messmate when it was first introduced suggests that they did not have a strong pre-existing behavioural aversion to eating messmate. Instead, the decline in messmate intake over the first three days post-introduction suggests that the koalas developed an aversion, potentially through post-ingestive feedback [49, 50]. The finding that individual koalas differed in their initial acceptance of messmate and that this influenced intake post-inoculation also suggests that the koalas may have differences in prior experience, genetics or physiology that affect their willingness to feed on messmate. Most plant secondary metabolites are detoxified in the liver and variation in detoxification ability is seen between individuals [40]. Individual animals can also differ strongly in their metabolic rate and in their energy requirements [32]. Such differences could be responsible for the observed differences in preference. Therefore, diet selection in this species is likely to be determined by a complex association between the koala’s physiology and GI microbiome.

Our development of faecal inoculations that are able to successfully introduce and establish enteric microbes into the GI tract of koalas provide a tool that could have wide applications for koala conservation. Koalas are listed as vulnerable by the Australian Federal Government and face differing challenges over their geographic range. In north eastern Australia, koalas have recently declined due to habitat loss and disease [51, 52]. Large numbers of koalas are brought into rehabilitation clinics each year [53] and are often treated with antibiotics that can cause gastrointestinal dysbiosis [54]. Our orally administered faecal inoculation capsules could be further developed for use as probiotics in such koalas to prevent dysbiosis and restore functional GI microbiomes. In southern Australia many populations of koalas have become overabundant [55, 56] and some are translocated to reduce local densities [55, 57, 58]. Faecal inoculations could be adapted for use in such instances to assist the koalas’ GI microbiomes in adapting to the dietary tree species available at the release sites or to assist koalas to shift diets in situ, preventing the need for translocations. However, such applications would require further refinement of the capsules for increased longevity and production efficiency.

## Conclusion

This study has revealed that GI microbiomes may not always adjust to a dietary change within an individual, even if such adaptation exists in the species or population as a whole. Limited horizontal transmission of particular microbial species may constrain the dietary niche of individual animals leading to dietary separation within a species due to differing digestive abilities of their symbiotic GI microbiomes. Such niche partitions have significant ramifications for a species’ ecology and habitat selection.

## Methods

### Study design

Twelve wild-caught koalas were captured from mature manna gum (*E. viminalis*) forest over a 3.5 km^2^ area at Cape Otway (38° 50′ 8.07“ S, 143° 31’ 7.00” E) and brought into captivity at the Conservation Ecology Centre, Cape Otway, Victoria (Additional file 2: Figure S2; Additional file 3: Table S1). Koalas were captured in two cohorts of six koalas (3 male and 3 female). The first cohort was held between October and December 2017 and the second was kept from January to March 2018. See Additional file 2: Figure S3 for an outline of the study design.

The koalas were allowed to habituate to their enclosures for a minimum of four days before being administered with 400 mg per kg (of body weight) of Cobalt ethylenediamine tetraacetic acid (Co-EDTA) and 800 mg per kg of Chromium-mordanted plant fibre particles (500–1000 μm). Faeces sampled were collected for 14 subsequent days, to determine the retention time of these inert digestion markers by koalas (manuscript in preparation). During this time the koalas were exclusively fed manna gum foliage.

After the completion of the retention time study, messmate foliage was introduced to the koalas and all koalas were offered both messmate and manna gum foliage for a period of three nights in the case of cohort 1 and eight nights for cohort 2. This difference between the cohorts was solely for unavoidable logistical reasons. Nonetheless, the GI microbiomes of both cohorts were sampled three days after the introduction of messmate (see below).

The koalas were then assigned to either the control or treatment group (3 koalas per group in each cohort; 3 females and 3 males per group across the two cohorts) and administered a daily dose of faecal inoculum for a period of 9 d. A duration of 9 d was selected due to the unusually long passage rate of solute digesta markers in koalas (which is thought to be indicative of microbial retention times). Cork and Warner [59] measured mean retention time of solute associated Cr-EDTA in captive koalas to be approximately 9 d, while Krockenberger and Hume [32] found mean solute retention time to be 4.5 d in wild koalas, a difference attributed to the wild vs captive contrast. As the koalas in this study were housed in captivity, it was conservatively anticipated that 9 d of inoculation would be required to ensure the koalas’ entire intestinal fill was inoculated.

For the treatment koalas, the inoculum was sourced from wild, radio-collared koalas feeding on messmate foliage (see donor koalas section below). Control koalas were inoculated with mixed faecal material from themselves and the other control koalas in that cohort. The control replicated the effect of dosing and the potential disruption to the established GI microbiome from exposure to exogenous microbes.

The koalas were retained in captivity for a further 18 d post-inoculation, providing for a 9 day ‘wash-out’ period during which microbes that failed to establish would be eliminated from the koalas’ gastro-intestinal tracts and 9 subsequent days to assess koala feeding behaviour and GI microbiomes post-establishment. Throughout these 18 days, only messmate foliage was made available to koalas between 20:00 and 08:00 when most koala feeding occurs [60]. Both messmate and manna gum foliage were made available during the day to ensure that those koalas unwilling or unable to eat messmate could meet their nutritional needs. A second digesta marker dose was administered 4 d after the last inoculation for the retention time study.

The composition of the koalas’ GI microbiomes was tracked over the course of the experiment. Faecal samples were collected for microbiome analysis at: 1) capture; 2) immediately prior to the first introduction of messmate; 3) three days after the introduction of messmate; and 4) immediately; 5) nine; and 6) 18 d after the final inoculations. The feeding preferences of the koalas were tracked throughout the study by measuring the koalas’ daily diurnal and nocturnal intakes of messmate and manna gum (see below). The koalas were released at the point of capture after the completion of the experiment, with the exception of one control koala that was released 9 d after the completion of the inoculations due to an unacceptable level of weight loss (> 10% of capture weight). All other koalas generally maintained weight over the coarse of the experiment after an initial drop during the first week in captivity (Additional file 2: Figure S4).

### Captive koalas

Koalas were captured using a standard noose and flag technique [61]. The koalas were anesthetised after capture and restrained with isoflurane (Isoflurane, Delvet Pty, Ltd.) in order to assess their health, age, sex and to establish if they possessed pouch young. None of the animals used in the study had pouch young or had any observable illness or injury. The majority of females on the Cape Otway Peninsula had previously been fitted with contraceptive implants (Levonorgestrel; Elorn Projects Pty Ltd., Southport, Queensland, Australia) in an effort by the Victorian Government to control koala overabundance at this site. We preferentially selected contracepted female koalas to minimise the chance of including pregnant koalas in the study. All selected koalas were in good condition (condition score ≥ 7) [62] and exhibited tooth wear from classes 3 to 5, corresponding to ages from 4 to 12 years [63].

### Husbandry

The koalas were housed individually in 2 × 2.5 m yards. Each yard was equipped with two resting forks and two feeding stations, with a 2 × 1.5 m area beneath the resting forks covered in artificial turf. The remaining floor area consisted of grass. Part of each yard was sheltered from the sun and rain with shade cloth. All *Eucalyptus* foliage provided to the koalas was cut as large branches from manna gum and messmate trees on Cape Otway. The branches were kept in water at all times to reduce desiccation. The koalas were provided water ad libitum. Faecal pellets were cleared from the artificial turf and branches daily.

### Donor koalas

Healthy adult koalas occupying patches of messmate trees (*E. obliqua*) on Cape Otway (Additional file 2: Figure S2) were used as faecal donors for the captive treatment koalas (Additional file 3: Table S1). At the beginning of the study, these koalas were captured, fitted with VHF radiotransmitter collars and released at the point of capture. The koalas were then radio-tracked over a period of at least 2 weeks prior to the faecal inoculations, as well as for 2 to 6 months during and after the faecal inoculations. The koalas’ daytime locations and resting tree species were recorded during this period on between 26 and 78 occasions.

Five koalas each were used as donors for cohort 1 and cohort 2. Two females and three male koalas were used as donors during cohort 1. Two of these koalas were observed in messmate stringybark (*E. obliqua*) trees on at least 78% of occasions, while a further two koalas were found in messmate on 50 and 61% of occasions. When these koalas were not observed in messmate they were generally found in manna gum, although they were occasionally found in non-food trees (0–14% of occasions), such as blackwood (*Acacia melanoxylon*). The fifth koala was often found in non-food trees and on the ground (18% of occasions) and was found in messmate on 63% of occasions, however, this increased to 76% of occasions when ground/non-food tree locations were excluded. Three of the same koalas were used as donors during cohort 2. However, the two koalas observed in messmate trees the least were replaced with two male koalas such that the donors for the second cohort consisted of one female and four males. The two alternative males were observed in messmate trees on 82 and 94% of occasions.

Several studies have concluded that changes in the faecal microbiome community structure are minimal within the first 24 h post production [64–67]. Therefore, faecal collections for the preparation of the treatment inoculum were made by locating the donor koalas in the late afternoon (typically after 17:00 h) on each day prior to an inoculation day. The donor koalas’ faecal pellets were collected overnight by placing a shade cloth sheet on the ground beneath the koala. The pellets were then collected from the drop-sheet early the following morning and using gloves, placed into a plastic zip-lock bag, with fresh inoculum processing commencing within 1 h of collection. Subsamples of the donor koalas’ pellets were retained for microbiome analysis.

At the conclusion of the faecal collection periods the koalas were captured and their collars were removed before release at point of capture.

### Faecal inoculations

Captive koalas in both cohort 1 and 2 were inoculated with two full capsules containing fresh, processed faecal material each day of the 9-day inoculation period. In addition to this, koalas in cohort MG2 received 1–2 capsules containing dried ground faecal material per day (produced a week prior to the inoculations). Koala faecal microbial communities are known to differ from those higher in the gastrointestinal tract [17]. However, faecal material was the only practical/ethical source of GI microbes for this study and has been successfully used in other studies [8, 30].

The capsules were administered to the koalas using a ‘pill popper’ while they were restrained in a hessian sack by an investigator. Administration of the capsules took less than 5–10 min per koala (including capture time). Antibiotics were not administered to the koalas as antibiotics do not necessarily improve the establishment of inoculated microbes and can result in a lasting reduction in microbial diversity [31].

To prepare the fresh capsules for administration, fresh pellets from up to five messmate donor koalas were pooled (Additional file 3: Table S2). Fresh pellets collected from the captive control koalas were also pooled separately. The faecal inoculum was then prepared according to a modified protocol from Hirsch et al. [30]. The faecal pellets were mixed with ¼ Ringer’s solution and worked into a slurry, first by manual manipulation and then using a vortex mixer. The slurry was then centrifuged for 15 min and the supernatant removed by pipetting. Three layers of pellet typically formed: a lower pellet of large fibrous fragments; a fine particle layer and a white layer that was assumed to be bacterial (Additional file 2: Figure S1). The upper fine particle and bacterial layers were collected, pooled and centrifuged in a Clements (GS 150) centrifuge at 3 700 rpm for 60 min. The supernatant was then removed and the resulting pellet mixed for use as inoculant. The inoculant was dispensed into size 0 acid-resistant hypromellose capsules (DRCaps, Capsugel®). The capsules were then banded with a shellac solution (37% w/v shellac, 61.5% v/v ethanol and 1.5% v/v Tween 20) and subsequently placed within a size 00 acid-resistant hypromellose capsule, which was then thinly coated in the shellac solution. The capsules were left to dry (for approximately 15 min) and then administered within an hour to the koalas.

Prior to the faecal inoculation study, we tested the performance of these capsules in vitro and showed that bacteria can survive within the capsules in the presence of acid (pH 1.7–1.9) for greater than 10 h (exceeding the transit time of digesta in a koala’s stomach; [15]) and that the capsules degrade when placed in a neutral solution similar to the environment of the mid and hindgut (Additional file 1). Samples from each of the pellet layers as well as the final inoculant were collected for microbiome assessment.

To prepare the dry capsules for administration, faeces from the control and treatment donors was air dried at ambient temperature in a brown paper bag for 24–48 h. The pellets were then ground into a fine powder using a domestic coffee grinder. The powder was then packed into Size 00 acid-resistant hypromellose capsules and thinly coated with shellac solution. The dried faecal material was administered to the koalas within 2 weeks of production.

### GI microbiome assessment

A total of 98 faecal samples were collected for microbiome assessment. This included 70 samples from the captive koalas over the course of the study, ten samples from donor koalas, two from other koalas found in messmate forest, six from other koalas located in manna gum forest (including one that become a captive koala in MG2), three treatment inoculant samples and seven samples from layers formed during centrifugation of the donor faecal samples (three from a single donor koala and four from a mixed sample). The full set of six samples were collected from ten of the captive koalas. We were unable to collect the final sample from the control koala released early (ID:B) and the capture sample from a treatment koala (ID = G) was lost during transport.

For each faecal sample, total genomic DNA was extracted from approximately 50-70 mg of material taken from the centre of a faecal pellet. The material was beaten for 5 min at 2,000 rpm using the MoBio PowerLyzer24 in a MoBio bead tube containing 0.1 mm dia. Zirconian/silica beads and 750ul of TLA buffer (Promega). The samples were centrifuged at 10,000 g for 30 s. DNA was then extracted from 150 ul of the supernatant using the Maxwell 16 robotic system and corresponding Tissue DNA kit (Promega) following the manufacturer’s instructions. Negative controls were included for each extraction kit.

A 589 bp section of the 16 s rRNA gene (V5 – V8 region) was amplified using 803F and 1392R primers [68] from the DNA extracts following the workflow outlined by Illumina (#15044223 Rev.B) except that Q5 Hot Start High-Fidelity 2X Master Mix (New England Biolabs) was used. PCR products were indexed with unique 8 bp barcodes using the Illumina Nextera XT 384 sample Index Kit A-D (Illumina FC-131-1002). Indexed amplicons were isolated using Qiagen QIAquick Gel Extraction Kit, as per manufacturer’s instructions. Paired-end sequencing was performed at the Australian Centre of Ecogenomics, on the Illumina Miseq using the version 3 reagent kit for 300 cycles.

Forward reads were processed and assigned taxonomic designations by QIIME 2 (v. 2017.10; [69]) using the SILVA 128 database [70, 71]. The resulting microbial feature by sample table was rarefied to 10 000 reads per sample using the vegan package [72] in R (version 3.5.0; [73]). All community composition analyses were performed on the rarefied dataset. Microbiome richness was estimated by a count of unique features recovered per sample after rarefaction and by calculating the Chao Index of alpha diversity using the package fossil in R [74]. Microbiome diversity within koalas was estimated using the Shannon diversity index as calculated using the vegan package. Weighted Unifrac distances between samples were calculated in QIIME 2. Variation in the relative abundance of the microbial groups was described by principal coordinate analysis (PCoA) generated from the weighted Unifrac distances using the vegan package. Significant differences between groups in microbial composition were determined by PERMANOVA from the weighted Unifrac distances using the vegan package. Other differences were tested using linear regression models, t-tests and the Wilcoxon rank sum test, where the residuals did not conform to normality.

Indicator species analysis was conducted using the indicspecies package in R [75] to determine which microbial features were significantly associated with a messmate diet when compared to a manna gum diet. All koalas found in manna gum forest (excluding the koala that later become a captive koala; *n* = 5) and the captive individuals at capture (with the exception of two captive koalas with GI microbiomes that did not resemble that of typical manna gum koalas; *n* = 9; see results: success of the faecal inoculations), were included in the manna gum group for the analysis. A single sample from each treatment donor (with the exception of the koala that was only found in messmate on 50% of occasions; *n* = 6) and two other koalas found in messmate forest were included in the messmate group. This analysis returned two statistics for each identified feature (indicator species): A = the probability that a sample came from a messmate koala given the feature was found; and B = the probability of finding that feature in a messmate koala. We used the suggested parameters in the indicspecies package documentation and considered microbial features for which A exceeded 80% and B exceeded 35% to be true indicator species.

### Diet assessment

We measured dry matter intake (DMI) of messmate and manna gum foliage by weighing branches immediately before they were offered to koalas, and immediately after they were removed to give a gross fresh matter intake. Gross wet matter intake was corrected for the change in mass of control branches that were kept outside koala pens. A sample of foliage from each control branch (20–60 g) was dried for 24 h at 80 °C to calculate the dry matter (DM) content of foliage and this was used to calculate the gross DMI of koalas. Koalas also regularly dropped leaves onto the ground while they were feeding. These leaves were collected each time branches were removed from the pens, identified as messmate or manna gum, dried and weighed. The dropped leaves’ dry weight value was subtracted from the gross DMI for each tree species to give the actual DMI.

We calculated average daily intake (ADI) for each koala prior to the introduction of messmate, when the koalas were feeding exclusively on manna gum. This provided a measure of the individual differences in maintenance energy requirements between the koalas due to differences in body size as well as digestive and metabolic efficiency. We also calculated the night time DMI of messmate for each koala prior to the faecal inoculations. This provided a measure of each koala’s individual willingness to feed on messmate prior to experimental manipulation. We then determined the night-time DMI of messmate for each koala during and after the faecal inoculations. For all of these calculations, feeding periods (12 h windows) in which the control branches increased in weight by more than 10% or decreased by more than 5% were excluded because changes beyond these limits introduced increased error into our estimations of DMI. Branches generally increased in weight when water collected on the leaves during rain and this was seen to be a consistent change across branches. Branches typically lost weight if the leaves lost moisture when exposed to sun and wind. These changes were observed to be somewhat idiosyncratic between branches and so a more stringent limit was imposed for weight loss than gain. These limits meant that a large proportion of day time intake measurements were excluded such that total intake and manna gum intake (that was fed only during the day during most of the experiment) could only be reliably assessed on a subset (52%) of days. However, our estimates of night-time intake, when messmate was primarily consumed were reliable on the vast majority of days.

#### Total dry matter intake

We used linear mixed models to assess the factors affecting total daily dry matter intake over the experiment. The models were constructed in R using the package lme4 [76] with the significance of the fixed effects calculated using the package lmerTest [77]. Koala ID was included as a random effect in all analyses to account for the repeated measures study design. The date was also included as a random factor. The phase of the experiment, treatment group and the change in the koalas’ GI microbiomes over the course of the experiment were considered as explanatory variable in separate models. To allow for the possibility that intake may have changed over time in the treatment group and in koalas that showed a change in their GI microbiomes, we also included the number of days after the first faecal inoculation was administered as an interaction term with the experimental group (treatment/control) and GI microbiome change explanatory variables. Non-significant variables were removed by backward elimination and all reported *p* values were taken from the final model.

#### Intake of messmate prior to the inoculations

We also used linear mixed models to assess the factors affecting night-time messmate intake over the first three days after its introduction and prior to the inoculations. We determined if intake of messmate differed between koalas, over time or if it could be explained by the composition of the koalas’ GI microbiomes. The koalas’ GI microbiomes at capture and immediately prior to the introduction of messmate were fitted as predictors in separate models. The Bacteroidetes to Firmicutes (B:F) ratio and the position of the koalas’ GI microbiomes on the first axis of the PCoA based on the weighted UniFrac distance matrix were used as measures of the koalas’ GI microbiome compositions. Manna gum ADI and cohort (1 or 2) were included as fixed co-variates in the analyses. The date was also included as a random factor to account for variation in environmental conditions, such as weather, that may have influenced feeding. Non-significant variables were removed by backward elimination.

#### Intake of messmate during and after the inoculations

Linear mixed effects models were also used to assess how messmate intake was influenced by the faecal inoculations and whether it was associated with the koalas’ GI microbiomes. Night-time messmate DMI was considered the best response variable due to its reliable estimation (see above), however, a subset of analyses were also performed using the proportion of total daily intake that was messmate as the explanatory variable to confirm our findings. Manna gum ADI, pre-inoculation intake of messmate and cohort were included as fixed co-variates in the night-time messmate DMI analyses. Koala ID was included as a random effect in all analyses to account for the repeated measures study design. The date was also included as a random factor.

A series of potential explanatory variables were assessed in separate models: 1. whether an koala belonged to the treatment or control group; 2. the koalas’ GI microbiomes at capture (B:F ratio and PC1 score); 3. the koalas’ GI microbiomes immediately prior to a phase; 4. the koalas’ GI microbiomes immediately after a phase; and 5. the overall change in the koalas’ GI microbiomes over the course of the experiment. The Akaike information criterion (AIC) was used to assess whether the GI microbiome prior to or after a phase was a better predictor of messmate intake when both variables were significant. To allow for the possibility that messmate intake may have changed over time in the treatment group and in koalas that showed a change in their GI microbiomes, we also included the number of days after the first faecal inoculation was administered as an interaction term with the experimental group (treatment/control) and GI microbiome change explanatory variables. We modelled the entire period during and post-inoculation combined as well as the different phases separately (i.e. during, washout and post-establishment). Non-significant variables were removed by backward elimination and all reported *p* values were taken from the final model.

## Additional files

Additional file 1: In vitro development of capsules for faecal inoculation experiment. (PDF 89 kb)

Additional file 2: **Figure S1.** Image of the layers formed when the faecal samples were mixed with ¼ Ringers solution to form a slurry and centrifuged for 15 min. **Figure S2.** Map of study site indicating koala capture locations, eucalupt forect type and site of location of captive experiement. MG1 = captive koala cohort 1, MG2 = captive koala cohort 2, MM = treatment donor koalas. **Figure S3.** Timeline for faecal inoculation experiment. Blue circles indicate when faecal samples were collected for GI microbiome assessment. In the green feeding schedule bar, MG = manna gum; and MM = messmate. D = Day, N=Night. Weights of the captive koalas over time. Experiment day 0 was the first day of the faecal inoculations. The inoculation period is indicated by the grey box. Each koala is represented by a different symbol. Control koalas are shown in blue and treatment koalas are shown in red. The control koala shown by the open square was released 9 days after the faecal inoculations concluded due to unacceptable weight loss. (PDF 393 kb)

Additional file 3: **Table S1.** Study Koalas. **Table S2.** Donor Koala Faeces Used in Inoculum on Each Day: Cohort MG1. **Table S3.** Donor Koala Faeces Used in Inoculum on Each Day: Cohort MG2. (PDF 428 kb)

## Data Availability

Sequencing data are available in the NCBI SRA database (Bioproject: PRJNA554505) and processed datasets are available in Dryad (10.5061/dryad.45ct519).

## Abbreviations

ADI: Average Daily Intake: referring to the average dry matter weight of *Eucalyptus* foliage consumed by a koala in a 24 h period
DMI: Dry Matter Intake: The amount of *Eucalyptus* foliage consumed as measured by dry weight
GI: Gastrointestinal microbiome: The microbial community associated with the gastrointestinal tract of an animal
TDMI: Total Dry Matter Intake over a 24 h period
