# Supplementary material for: Faecal inoculations alter the gastrointestinal microbiome and allow dietary expansion in a wild specialist herbivore, the koala
Source: Anim Microbiome. 2019 Aug 21;1:6. doi: 10.1186/s42523-019-0008-0 (PMC7803123; doi:10.1186/s42523-019-0008-0)
Supplement: Supplementary file 1 — In vitro development of capsules for faecal inoculation experiment. (PDF 89 kb) [file 42523_2019_8_MOESM1_ESM.pdf]

Online Supplement 1: In vitro development of capsules for faecal inoculation experiment

Aims

1. Develop a capsule that would remain intact for 10 h in an acid solution and that would breakdown within 3 h of transfer into a neutral/alkaline solution.
2. To assess bacterial survival in the selected capsule for 10 h in an acid solution.

Methods

Capsule Configurations

Three capsule configurations were tested. The first configuration (**bare capsules**) consisted of only acid-resistant hypromellose capsules (DRCaps, Capsugel®). In the second configuration (**banded capsules**) shellac solution (37% w/v shellac, 61.5% v/v ethanol and 1.5% v/v Tween 20) was applied around the join between the two halves of the capsules and left to dry. For the third configuration (**coated capsules**) the shellac solution was not only applied to the join between two capsule halves but was also applied to the entire surface of the outer most capsule. For the coated capsule configuration, care was taken to leave gaps in the coating to allow the neutral/alkaline solution to come into contract with the capsule surface (see results). Each capsule configuration was tested with one, two and three layers. Single layer capsules consisted of a size 00 capsule. The two layer capsules consisted of a size 0 capsule nested within a size 00 capsule, while, the three layer capsule also had a size 1 capsule nested within the size 0 capsule.

Capsule Performance Trials

To test the performance of the different capsule configurations and number of layers, three replicate capsules were prepared for each configuration/layer combination. The capsules were filled with a mix of glycerol, saline and crystal violet. The capsules were placed in a 250 ml conical flask of an acid solution containing 2g l^{-1} sodium chloride, 3.2g l^{-1} of pepsin and hydrochloric acid such that the solution had a pH of 1.7-1.9. This solution was designed to mimic the acid environment present in a koala's stomach (15). The acid solutions containing the capsules were then rocked at 100 rpm at 37 °C. The capsules were visually inspected every hour for a maximum of 10 h to assess their integrity. The presence of the crystal violet

within the capsules allowed for rapid visual identification of when a capsule had been compromised, as the acid solution rapidly became coloured.

If the capsules remained intact for 10 h then they were transferred into a 250 ml conical flask of alkaline solution containing 3g l^{-1} bile salts and sodium hydroxide such that the solutions had a pH of 6.8. This solution mimicked the environment present in the koala hindgut (15). Again, the solutions containing the capsules were rocked at 100 rpm at 37 °C. The capsules were visually inspected every 30 min until they became compromised.

Bacterial Survival

To assess the survival of enteric bacteria within the selected capsule configuration, caecal contents were harvested from a euthanized koala at the Port Macquarie Koala Hospital within 30 min of death. The contents were then kept at 10 °C for 48 h prior to processing as described in the main text for the faecal pellets of the donor animals.

The processed caecal contents were then dispensed into 20 capsules. The capsules were then incubated in the acid solution as described for the capsule trials. Every hour, two capsules were removed from the acid solution. The contents of the capsules were diluted ($1:10^5$) in ¼ Ringers solution and 40 µl of the dilution was then spread onto an LB agar plate (Power and McCuen, 1988) and onto a Wilkins-Chalgren Anaerobe agar plate (WCAA; Oxoid). The LB plates were incubated aerobically at 37 °C for 24 h. The WCAA plates were incubated anaerobically in a BD GasPak™ EZ Large Incubation Container at 37 °C for 72 h. The numbers of colony forming units were then determined by manual count.

Results and Conclusions

Capsule Performance Trials

Visual inspection of the capsules in the acid solution showed that all the one and two layer bare capsules became compromised within one hour, with crystal violet visible in the acid solution. Crystal violet was not visible in the solution of the three layer bare capsules until three hours. In all cases the bare capsules failed because the two halves of the capsules came apart.

In the case of the banded capsules, the single layer capsules failed after three hours in the acid solution, while, the two layer capsules failed between three and four hours. The replicates of the three layer banded capsules varied in their longevity. One capsule failed after just three hours, the second remained intact for seven hours and the third failed just prior to the completion of the 10 h experiment. In all multilayer capsules it was evident that the inner

capsule failed first, prior to the external capsule. This was assumed to be due to the neutral liquid present inside the capsule degrading the acid-resistant material.

By contrast, the single layer coated capsules remained intact for 6 h in the acid solution, while, both the two and three layer coated capsules remained intact for 10 h. The two and three layer coated capsules were deformed in shape by the end of the 10 hour treatment and the shellac coating had become opaque in colour. However, no trace of crystal violet was present in the acid solution. The two and three layer coated capsules were compromised within 2 h of being transferred into the alkaline solution. However, it was noted that the shellac coating remained intact and that the contents were only able to exit the capsule where the coating was absent. Therefore, the two layer coated capsules were selected for the bacterial survival experiment.

Bacterial Survival

The aerobic and anaerobic bacterial cell densities did not increase or decrease over the 10 hour period that the capsules were incubated in the acid solution (aerobes: $F = 0.042$, $p = 0.841$; anaerobes: $F = 1.194$, $p = 0.289$; Fig S5). Considerable variation in cell densities was seen between time points. However, this change was within an order of magnitude and likely reflected cell density heterogeneity in the caecal contents used to fill the capsules. Therefore, it was concluded that live bacteria could be delivered to the hindgut of the koala using the two layer coated capsules. As such, these capsules were selected for use in the faecal inoculation experiment.

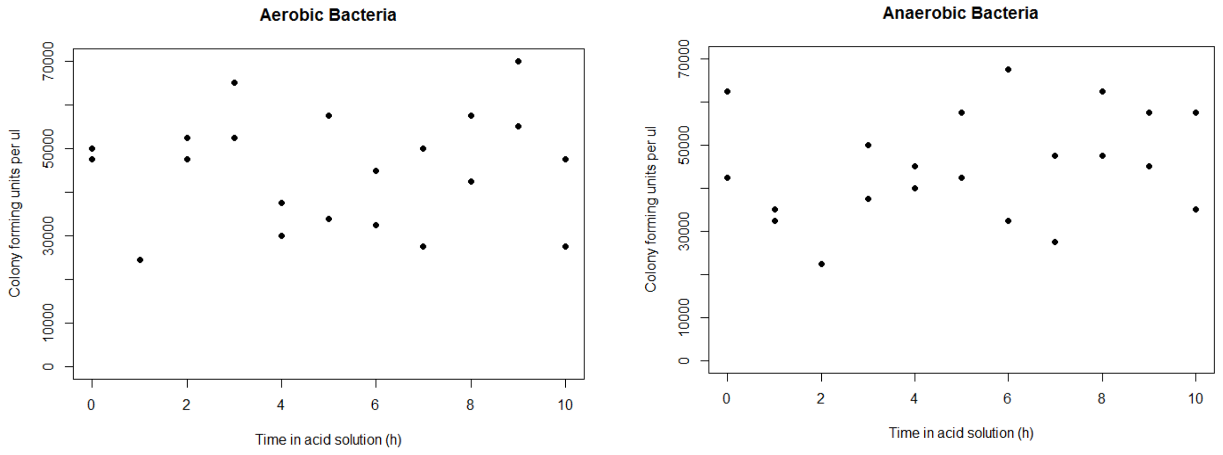

Fig S5: Survival of aerobic and anaerobic bacteria in capsules over a t10 h period when incubated in hydrochloric acid (pH of 1.7-1.9).
