# Supplementary material for: Faecal inoculations alter the gastrointestinal microbiome and allow dietary expansion in a wild specialist herbivore, the koala
Source: Anim Microbiome. 2019 Aug 21;1:6. doi: 10.1186/s42523-019-0008-0 (PMC7803123; doi:10.1186/s42523-019-0008-0)
Supplement: Supplementary file 2 — Figure S1. Image of the layers formed when the faecal samples were mixed with ¼ Ringers solution to form a slurry and centrifuged for 15 min. Figure S2. Map of study site indicating koala capture locations, eucalupt forect type and site of location of captive experiement. MG1 = captive koala cohort 1, MG2 = captive koala cohort 2, MM = treatment donor koalas. Figure S3. Timeline for faecal inoculation experiment. Blue circles indicate when faecal samples were collected for GI microbiome assessment. In the green feeding schedule bar, MG = manna gum; and MM = messmate. D = Day, N=Night. Weights of the captive koalas over time. Experiment day 0 was the first day of the faecal inoculations. The inoculation period is indicated by the grey box. Each koala is represented by a different symbol. Control koalas are shown in blue and treatment koalas are shown in red. The control koala shown by the open square was released 9 days after the faecal inoculations concluded due to unacceptable weight loss. (PDF 393 kb) [file 42523_2019_8_MOESM2_ESM.pdf]

Supplementry Figures

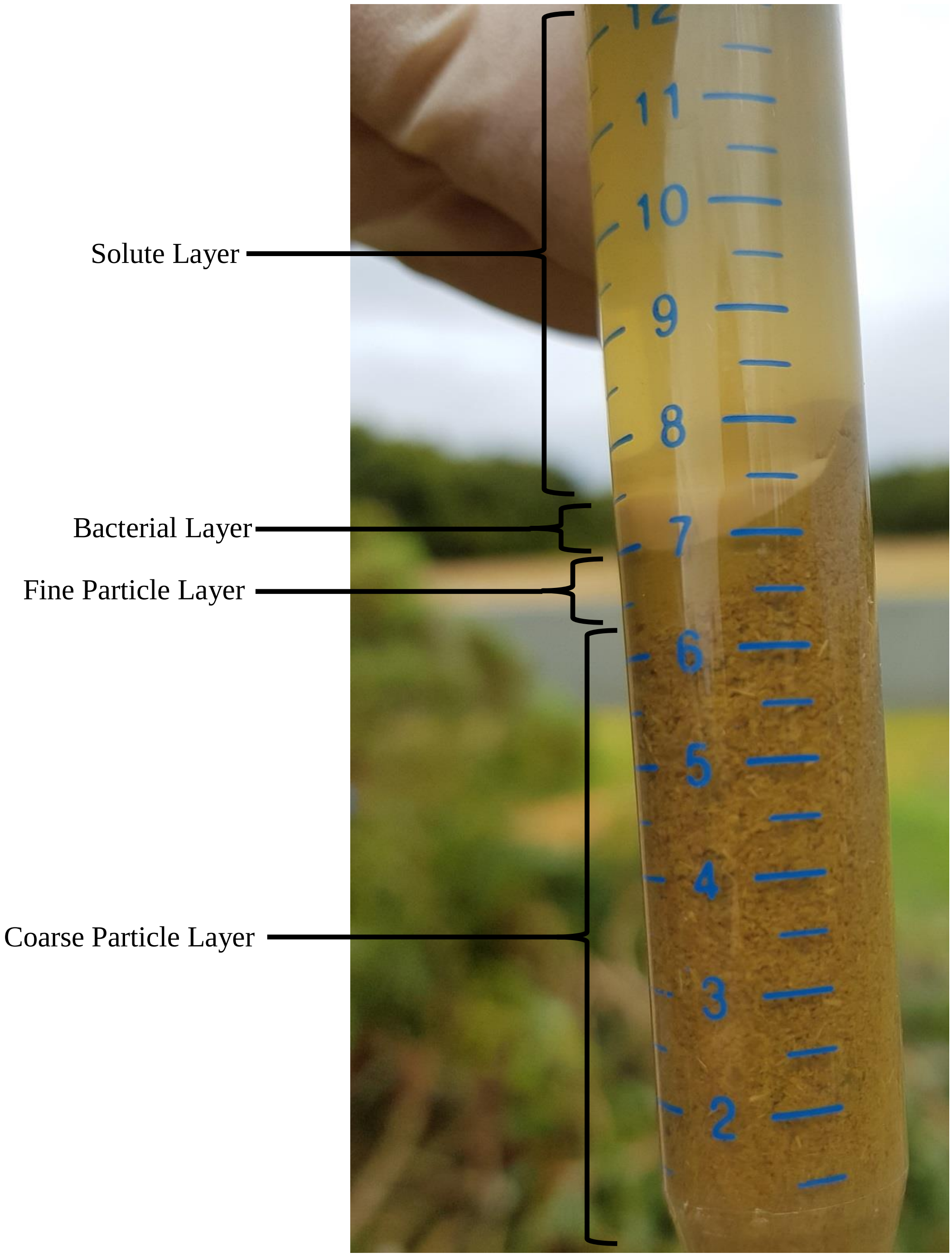

Fig S1: Image of the layers formed when the faecal samples were mixed with ¼ Ringers solution to form a slurry and centrifuged for 15 minutes.

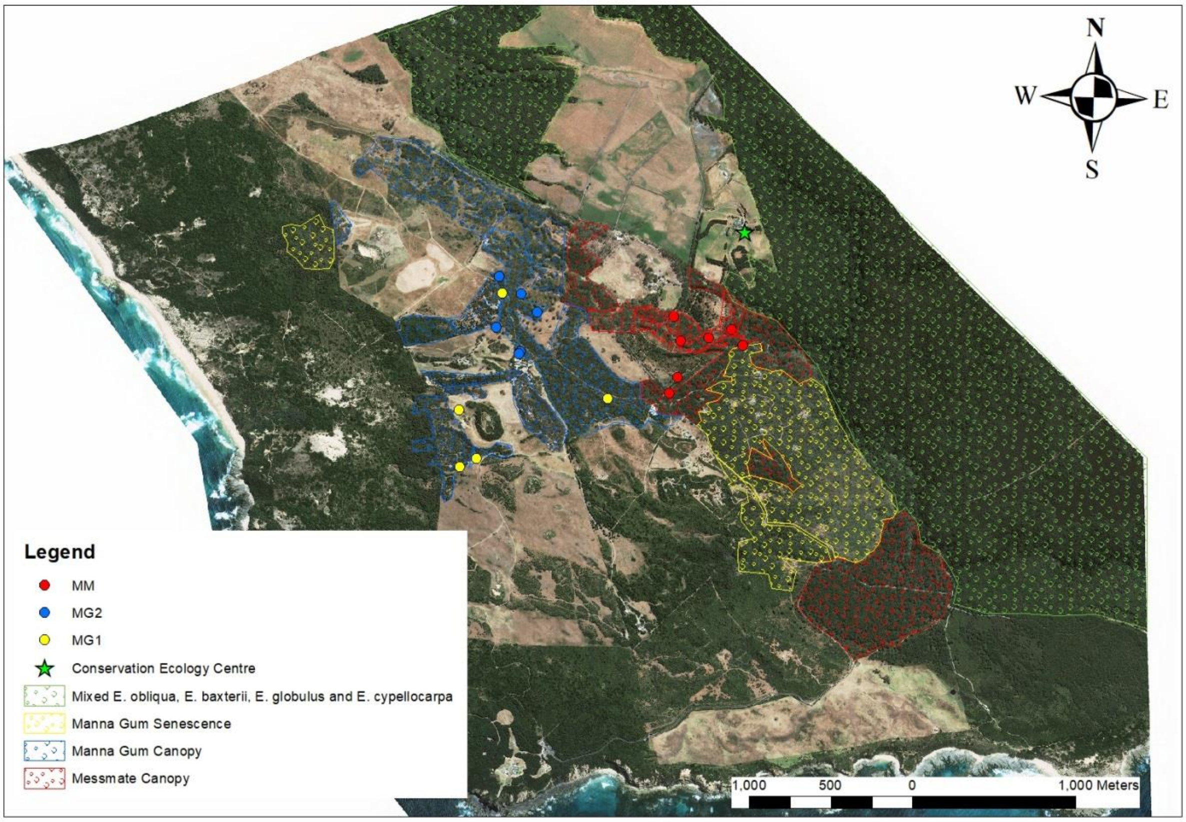

Fig S2: Map of study site indicating koala capture locations, eucalupt forect type and site of location of captive experiement. MG1 = captive koala cohort 1, MG2 = captive koala cohort 2, MM = treatment donor koalas.

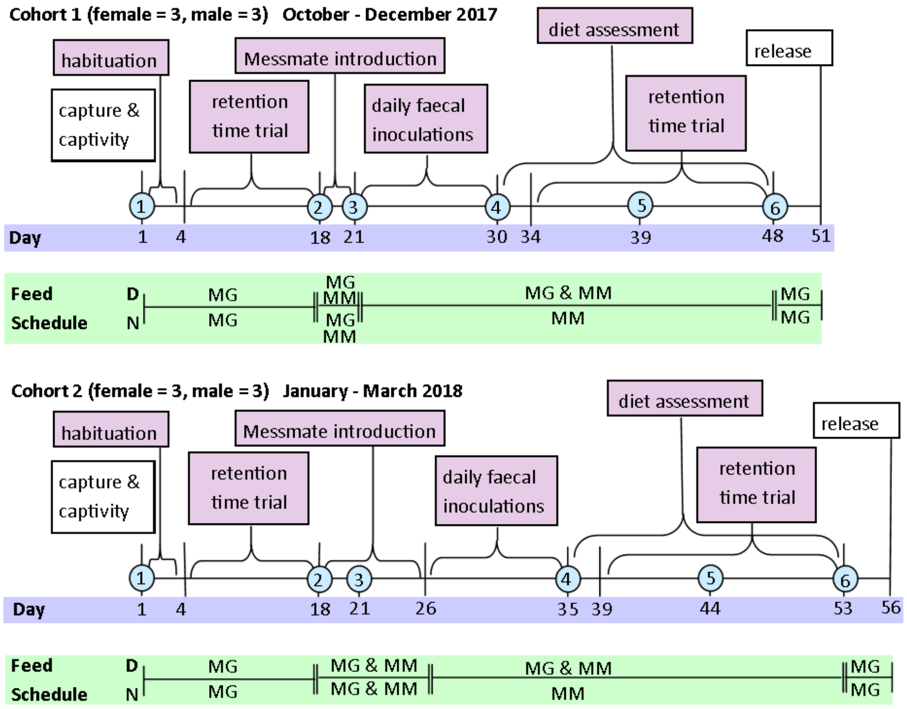

Fig S3: Timeline for faecal inoculation experiment. Blue circles indicate when faecal samples were collected for microbiome assessment. In the green feeding schedule bar, MG = manna gum; and MM = messmate. D=Day, N=Night.

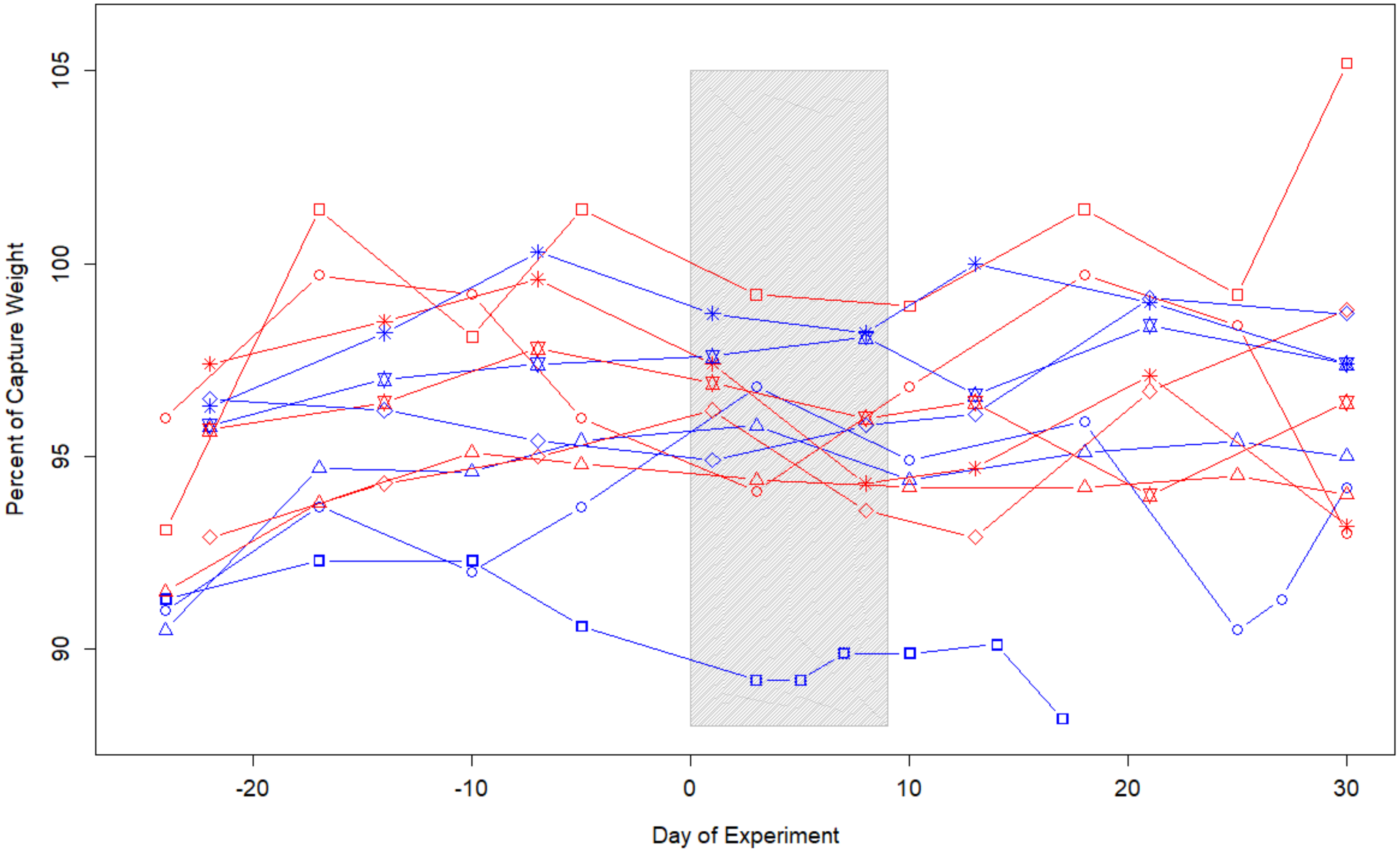

Fig S3: Weights of the captive koalas over time. Experiment day 0 was the first day of the faecal inoculations. The inoculation period is indicated by the grey box. Each koala is represented by a different symbol. Control koalas are shown in blue and treatment koalas are shown in red. The control koala shown by the open square was released 9 days after the faecal inoculations concluded due to unacceptable weight loss.
