# Supplementary material for: Faecal inoculations alter the gastrointestinal microbiome and allow dietary expansion in a wild specialist herbivore, the koala
Source: Anim Microbiome. 2019 Aug 21;1:6. doi: 10.1186/s42523-019-0008-0 (PMC7803123; doi:10.1186/s42523-019-0008-0)
Supplement: Supplementary file 3 — Table S1. Study Koalas. Table S2. Donor Koala Faeces Used in Inoculum on Each Day: Cohort MG1. Table S3. Donor Koala Faeces Used in Inoculum on Each Day: Cohort MG2. (PDF 428 kb) [file 42523_2019_8_MOESM3_ESM.pdf]

Supplementry Tables

Table S1: Study koalas

ID	Study Group	Radio-Tracked in Wild	Capture Location	Sex	Toothwear
B	Captive MG1: control	No	38°50'04.4", 143°31'08.0"	F	3
C	Captive MG1: control	No	38°49'44.0", 143°30'40.5"	F	3
F	Captive MG1: control	No	38°50'18.8", 143°30'30.9"	M	3
D	Captive MG1: treatment	No	38°49'44.0", 143°30'40.5"	M	3
E	Captive MG1: treatment	No	38°50'17.1", 143°30'35.0"	F	4A
G	Captive MG1: treatment	No	38°50'07.6", 143°30'30.3"	M	4A
U	Captive MG2: control	No	38°49'40.7", 143°30'39.6"	M	4C
V	Captive MG2: control	No	38°49'56.0", 143°30'45.0"	F	Not Recorded
W	Captive MG2: control	No	38°49'44.2", 143°30'45.2"	M	3
P	Captive MG2: treatment	Yes	38°49'47.6", 143°30'49.3"	F	left: 5, right: 4B
T	Captive MG2: treatment	No	38°49'55.6", 143°30'45.4"	F	4C
X	Captive MG2: treatment	No	38°49'50.9", 143°30'39.1"	M	3
L	Donor for MG1	Yes	38°49'53.2", 143°31'41.8"	M	4B
R	Donor for MG1	Yes	38°50'04.0", 143°31'15.1"	F	5
A	Donor for MG1 and MG2	Yes	38°49'59.3", 143°31'25.0"	M	4C
H	Donor for MG1 and MG2	Yes	38°49'49.9", 143°31'37.7"	F	4A
I	Donor for MG1 and MG2	Yes	38°49.899, 143°31'31.501	M	3
J	Donor for MG2	Yes	38°49'47.8", 143°31'24.2"	M	4B
S	Donor for MG2	Yes	38°49'54.1", 143°31'36.4"	M	4A
C	Wild Manna Gum koala	Yes	38°49'57.9", 143°30'55.4"	M	Right 6, left 7
M	Wild Manna Gum koala	Yes	38°49'52.1", 143°30'43.2"	F	4C
N	Wild Manna Gum koala	Yes	38°49'47.7", 143°30'41.2"	M	3
O	Wild Manna Gum koala	Yes	38°49'46.2", 143°30'44.6"	M	4A
Q	Wild Manna Gum koala	Yes	38°49'46.4", 143°30'46.3"	F	4B
Y	Wild Messmate Koala	Yes	38°49'57.8", 143°31'29.2"	F	4C

Table S2: Donor koala faeces used in inoculum on each day: Cohort MG1

ID	1	2	3	4	5	6	7	8	9
A	Y	Y	Y	Y	Y	Y	Y	Y	Y
H	Y	Y	Y	Y	Y	Y	Y	Y	Y
I	Y	Y	Y	Y	Y	Y	Y	Y	N
L	N	Y	N	Y	Y	Y	Y	N	Y
R	N	N	Y	N	Y	Y	Y	Y	Y

Y= faeces from that koala was included on that day, N = faeces from that koala was not included on that day

Table S3: Donor koala faeces used in inoculum on each day: Cohort MG2

ID	1	2	3	4	5	6	7	8	9
A	Y	Y	N	Y	N	Y	Y	Y	N
H	Y	Y	Y	Y	Y	Y	Y	Y	Y
I	Y	Y	Y	Y	Y	Y	Y	Y	N
J	Y	Y	Y	Y	Y	Y	Y	Y	Y
S	Y	N	Y	Y	Y	Y	Y	Y	Y

Y= faeces from that koala was included on that day, N = faeces from that koala was not included on that day
